# Exploring Challenges Related to Breast Cancer Chemotherapy Among Females in Pakistan: A Qualitative Study

**DOI:** 10.1002/cnr2.70381

**Published:** 2025-11-13

**Authors:** Rehana Sarwat, Muhammad Amir Hamza, Ayesha Azam, Bushra Batool Zahra, Muhammad Amer, Zakir Khan, Maryam Mahmood, Ali Ahmed

**Affiliations:** ^1^ Riphah Institute of Pharmaceutical Sciences Riphah International University Islamabad Pakistan; ^2^ Department of Pharmacy Abasyn University, Islamabad Campus Islamabad Pakistan; ^3^ School of Pharmacy and Biomolecular Sciences RCSI University of Medicine and Health Sciences Dublin Ireland; ^4^ Pharmacy Practice and Global Public Health, School of Health and Social Care University of Essex Colchester Essex UK; ^5^ Division of Infectious Diseases and Global Public Health, School of Medicine University of California San Diego (UCSD) La Jolla California USA

**Keywords:** breast cancer, chemotherapy, experiences, patient safety, qualitative study, side effects, women

## Abstract

**Background:**

With the increased rate of breast cancer affecting one in every nine women in Pakistan, breast cancer treatment, particularly chemotherapy, is often very challenging for patients.

**Aims:**

This study aims to explore chemotherapy related‐challenges among females in Pakistan.

**Methods:**

Semi‐structured, face‐to‐face interviews were conducted with 40 post‐chemotherapy females at the tertiary care setting in Islamabad, Pakistan, from February 2024 to April 2024, using a purposive sampling technique. Each 15–20 min interview was audio recorded in the national language (Urdu), transcribed, and analyzed using a thematic analysis approach.

**Results:**

The study included women with an average age of 47 years; most were married (*n* = 33), had primary education (*n* = 13), were unemployed (*n* = 35), and had a low family income (*n* = 36). Overall, participants reported hair loss (*n* = 35), darkened nails (*n* = 26), mouth sores (*n* = 24), weakness (*n* = 21), vomiting (*n* = 20), diarrhea (*n* = 15), constipation (*n* = 13), and various other effects. Participants experienced significant physical challenges, psychological impact, economic strain, and informational challenges. However, coping strategies such as alternative treatments, staying motivated, managing side effects, and support from friends and family helped patients effectively deal with their chemotherapy.

**Conclusion:**

Our findings highlight the need for financial support programs and government interventions to enhance the affordability of chemotherapy treatment. Furthermore, this study implies that healthcare professionals improve patient outcomes by counseling them about chemotherapy and its possible side effects before the treatment begins.

## 1 Introduction

Breast cancer is the most often reported cancer and the primary cause of mortality among women worldwide [1]. In 2022, about 2.3 million women were diagnosed with breast cancer, leading to 670 000 global deaths [2]. The overall incidence rate of breast cancer is growing in many low‐ and middle‐income countries (LMICs) in contrast to high‐income countries (HICs) [3]. Pakistan has the most significant rate of breast cancer in Asia. Breast cancer continues to grow as a widespread disease in women in Pakistan that influences significant numbers from both urban and rural populations annually [4]. In Pakistan, one in nine women struggles with the risk of having breast cancer in their entire life, and a large number of women die of breast cancer each year [5].

The primary treatments for breast cancer include chemotherapy, surgery, radiation therapy, targeted therapy, and endocrine therapy [6]. Chemotherapy is an effective treatment option for breast cancer, either alone or in combination with other therapies, and is recommended to start promptly after diagnosis [7]. Chemotherapy damages cancerous cells and, to some extent, the other cells in the human body [8]. The procedure of chemotherapy may extend the lifespan and increase the chance of survival of cancer patients; however, it can also lead to adverse side effects, resulting in various unpleasant feelings and complications for the patients and their families [9]. Chemotherapy has been linked to a variety of side effects, including gastrointestinal symptoms (nausea, vomiting, constipation, diarrhea, indigestion, heartburn), neurological impacts (neuropathy, insomnia, fatigue, fever, weakness, hypothermia), immune responses (allergic reactions, alopecia), cardiovascular issues (chest pain, hypertension, low red blood cell count, low neutrophil count), skin problems (dry skin, rash), and endocrine disturbances (weight loss, weight gain, watery eyes) [10]. Chemotherapy also leads to physiological, psychological, informational, social, and support system‐related challenges, resulting in a significant decrease in patients' quality of life [11].

Many studies have been performed to evaluate the experiences of chemotherapy among women [12, 13, 14, 15, 16]. Several have reported chemotherapy‐related side effects, including fatigue [17], nausea [18, 19], vomiting [20, 21], hot flashes [22], and hair loss [17, 23], which can lead to significant depression with negative emotions, stress, and anger [24, 25, 26]. However, care and support provided by healthcare professionals, friends, and family encourage patients to deal with chemotherapy side effects and maintain their health [14, 27]. Women also use various coping techniques, including maintaining hope, positive thinking, staying motivated, keeping busy [28], and facing challenges with courage, willpower, and strength to succeed in the struggle against breast cancer [29]. Most of the reported experiences are relevant to the Pakistani context; however, some may be specific and not documented in the available literature. Therefore, this study explores the experiences and challenging issues of chemotherapy faced by women from different geographic and cultural backgrounds in Pakistan that are unreported in the literature. The research aims to fill the gap by investigating factors within the Pakistani context across various populations. These findings assist policymakers and healthcare professionals in achieving better outcomes for the treatment and quality of life of patients with breast cancer.

## 2 Methodology

### 2.1 Study Design

The research study used a qualitative approach to investigate individuals' subjective experiences and the chemotherapy side effects they experienced. This design was adopted to gain new perspectives and provide in‐depth knowledge of a certain phenomenon, supporting researchers to collect data on the experiences of patients that could not be recorded by a quantitative method [30]. This study adhered to the standards of the COnsolidated criteria for REporting on Qualitative Research (COREQ) (Table S1) to report the research findings [31].

### 2.2 Study Setting

This study was conducted at the Pakistan Institute of Medical Sciences (PIMS), a tertiary care hospital in Islamabad, Pakistan [32]. This healthcare institution was selected due to its geographical location, which facilitates access for patients from various cultural backgrounds and regions of Pakistan [33]. The outpatient department (OPD), which sees a daily influx of 40 cancer patients seeking medical assistance and chemotherapy to 10–15 patients, provided a credible and significant setting for this study [34].

### 2.3 Participant Selection

The purposive sampling approach was implemented to select patients who met the study requirements. The inclusion criteria were specific, targeting female breast cancer patients aged 18 or older who had completed all their chemotherapy sessions, were interested in participating in an audio‐recorded interview, and were recruited during a follow‐up hospital visit, where respondents were invited to share their experiences during chemotherapy rather than post‐treatment. The researcher excluded individuals experiencing physical discomfort, hospitalization, or those unwilling to participate in an audio‐recorded interview.

### 2.4 Study Tool

After reviewing relevant literature [15, 16], a semi‐structured interview guide (Table 1) was created. Argumentative and cumulative approaches were used to validate the interview guide, resulting in a reliable guide [35]. The booklet consisted of open‐ended questions and suitable probes aligned with the study's objective. The guide was used to gather data from the participants, allowing them to share their personal experiences. The experts reviewed the preliminary draft of the interview guide and, after that, piloted it on four women who met the inclusion criteria to verify its reliability. Subsequently, the guide was adjusted based on the results of the pilot testing.

**TABLE 1:** Interview guide.

Introduction First, thank you so much for your time participating in this interview about chemotherapy‐related challenges, which we are now recording. When did you know about your disease? Can you describe your hesitation/situation after the diagnosis of cancer? Did you have any prior knowledge about chemotherapy when you found out that you would have to undergo chemotherapy treatment? Before the start of chemotherapy, did you experience fears or negative thoughts? How did you feel at that time? Did you receive support from friends, family, or healthcare providers during chemotherapy? If so, how were they dealing with you in managing the chemotherapy‐related difficulties? Did you use any alternative therapies other than chemotherapy? Did these therapies provide benefits? Did you experience financial barriers to chemotherapy treatment? How did you manage the expenses? Explain in detail any side effects you experienced because of chemotherapy? How have these side effects impacted your daily life, including social activities, family, and work? Describe how you manage or cope with physical or emotional side effects? Did you use any strategies that helped you manage them? Did you discuss your side effects and symptoms with your healthcare team? If so, then what advice or support did they provide? Could you share the most challenging aspects for you during chemotherapy? How did you deal with these challenges? How did you stay motivated during your chemotherapy treatment? Was there any source of courage that helped you to stay motivated? Closing Is there anything else you would like us to know about chemotherapy‐related challenges?

### 2.5 Data Collection

The first author (R.S.) approached each breast cancer patient who was coming up for follow‐up and had completed chemotherapy treatment and encouraged them to participate by explaining the study's purpose. They were allowed to participate voluntarily and withdraw from the study at any point. Individuals who fulfilled the inclusion criteria were provided with informed consent for signature. According to the agreed guide, semi‐structured face‐to‐face interviews were employed between February 2024 and April 2024. All interviews took place in Urdu, Pakistan's national language, and participants were assigned a particular identifying code (pseudonym) to ensure confidentiality and anonymity. Each interview was 15–20 min long and was recorded using an audio recorder after obtaining consent from the study participants. Transcriptions of the audio recordings from each interview were made. The interviews were initially transcribed in Urdu and subsequently translated into English. Key worries and impressions were documented during the interview by taking relevant notes. The interview process was conducted until saturation was achieved. Data saturation was operationalized as the point at which no new information is provided during the interview, and no new codes can be generated, leading to repetitive data [36]. After 38 interviews, data saturation was reached, and two additional interviews were conducted to validate the saturation further, confirming that no new information appeared. This strategy aligns with the saturation principle described by Saunders et al. [37].

### 2.6 Data Analysis

The audio recordings from each interview were used to transcribe data. During the interviews, relevant notes were also taken to document participants' nonverbal expressions. The interviews were transcribed in Urdu, subsequently translated into English, and verified further.

The transcripts of all the interviews were analyzed using thematic analysis, following the method defined by Braun and Clarke [38]. Initially, the researcher familiarized themselves with the data by repeatedly listening to and reading to interpret the transcribed data deeply. Subsequently, relevant data related to the research objectives were manually retrieved from all interviews on Microsoft Word, leading to the generation of initial codes. The researcher collated all the data and organized each similar and repeated code into a sub‐theme. Themes were developed by gathering multiple subthemes. The senior author evaluated the provided themes to ensure their validity and significance to the research topic. The final assessment made by the supervisor was declared significant. Further, all themes were given suitable titles. The final results included numerous direct narratives to ensure that the findings provided a direct and persuasive representation of the data.

### 2.7 Ethical Considerations

Approval to perform the research study was acquired by the Research Ethics Committee of Riphah Institute of Pharmaceutical Sciences, Riphah International University (Ref. No. REC/RIPS/2023/26) and PIMS Hospital Islamabad along with its affiliated educational institutions, Shaheed Zulfiqar Ali Bhutto Medical University (SZABMU) (No. F.1‐1/2015/ERB/SZABMU/1234). This study was done according to the ethical principles of the Declaration of Helsinki [39] and its subsequent revisions. Potential participants were provided with information regarding the objective and design of this study, and their informed consent was also gained. Furthermore, permission was obtained from each participant to record the interviews, and they have the right to withdraw their participation at any point.

## 3 Results

### 3.1 Participant Characteristics

A total of 40 women with an average age of 47 years (ranging from 30 to 70 years), with the most significant proportion of the participants being married (*n* = 33), having two or more children (*n* = 27), living in Islamabad (*n* = 19), receiving primary education (*n* = 13), being unemployed (*n* = 35), having a family income of ≤ PKR 50 000 per month (*n* = 36), having Stage II breast cancer (*n* = 20), having a family history of breast cancer (*n* = 14), and experiencing tumor recurrence (*n* = 6) (Table 2).

**TABLE 2:** Demographics.

Characteristics	*N* (%)
Age (years)	
30–49	25 (62.5%)
50–69	14 (35%)
≥ 70	1 (2.5%)
Marital status	
Single	2 (5%)
Married	33 (82.5%)
Widow	3 (7.5%)
Divorced	2 (5%)
Children	
0	6 (15%)
1	7 (17.5%)
≥ 2	27 (67.5%)
City/residence	
Islamabad	19 (47.5%)
Rawalpindi	4 (10%)
Others	17 (42.5%)
Education level	
Tertiary	10 (25%)
Secondary	6 (15%)
Primary	13 (32.5%)
None	11 (27.5%)
Employment status	
Employed	5 (12.5%)
Unemployed	35 (87.5%)
Family income (per month)	
≤ PKR 50 000	36 (90%)
> PKR 50 000	4 (10%)
Cancer stage	
I	6 (15%)
II	20 (50%)
III	10 (25%)
IV	4 (10%)
Family history	
Yes	14 (35%)
No	26 (65%)
Recurrence	
Yes	6 (15%)
No	34 (85%)

The most prevalent side effects of chemotherapy, as reported by patients in this study, included hair loss (*n* = 35), darkened nails (*n* = 26), mouth sores (*n* = 24), weakness (*n* = 21), and vomiting (*n* = 20) (Table 3).

**TABLE 3:** Chemotherapy side effects.

Side effects	*N* (%)
Body aches	10 (25%)
Constipation	13 (32.5%)
Coughing	1 (2.5%)
Darkened nails	26 (65%)
Diarrhea	15 (37.5%)
Dizziness	3 (7.5%)
Fatigue	3 (7.5%)
Feel thirsty	1 (2.5%)
Fever	5 (12.5%)
Hair loss	35 (87.5%)
Indigestion	8 (20%)
Insomnia	1 (2.5%)
Joint pain	6 (15%)
Loss of appetite	13 (32.5%)
Low HB	1 (2.5%)
Low WBCs	3 (7.5%)
Mobility issues	5 (12.5%)
Mouth sores	24 (60%)
Nausea	9 (22.5%)
Shortness of breath	1 (2.5%)
Skin darkening	5 (12.5%)
Swollen gums	1 (2.5%)
Taste alteration	10 (25%)
Toothache	3 (7.5%)
Vision problems	2 (5%)
Vomiting	20 (50%)
Weakness	21 (52.5%)
Weight loss	10 (25%)

### 3.2 Key Themes

**FIGURE 1:**
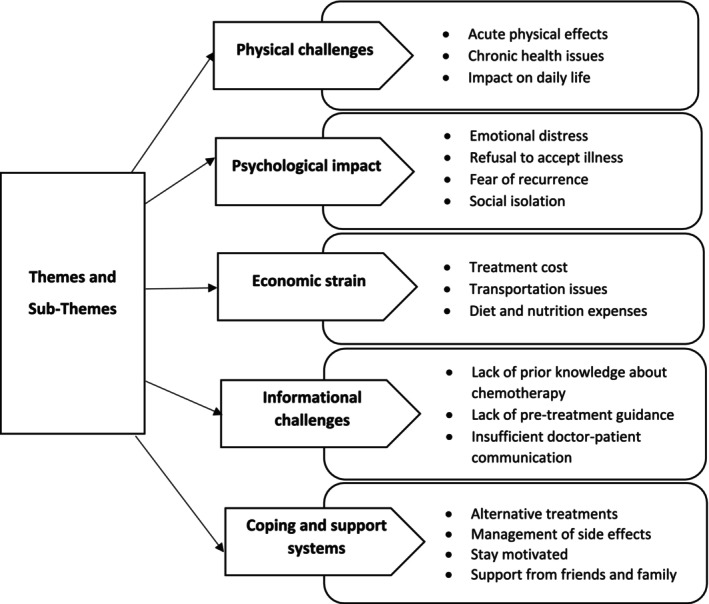
Patient experiences with breast cancer chemotherapy: themes and subthemes.

Thematic analysis generated five key themes: (1) Physical challenges, (2) Psychological impacts, (3) Economic strain, (4) Informational challenges, and (5) Coping and support systems. The themes were divided into several subthemes, which have been explained further, and patients' additional quotes are provided in Table S2. Figure 1 highlights the themes and subthemes that emerged from this study.

### 3.3 Theme 1: Physical Challenges

#### 3.3.1 Subtheme 1.1: Acute Physical Effects

Chemotherapy is a known cause of physiologic effects and is frequently encountered. Patients who have undergone breast cancer chemotherapy have complained of a range of side effects, including vomiting, nausea, diarrhea, and constipation. Several individuals reported the formation of blisters in their oral cavity, along with loss of taste sensation.

I also had side effects from the chemotherapy. I felt nauseous. These medicines also lead to constipation. Even now, if I drink less water, I get constipated. I did not want to eat or drink anything due to nausea. I vomited a lot. (P107, 49 years)

During chemotherapy, it became difficult for me to eat and drink. I had blisters in my mouth. As soon as food reached my stomach, I started vomiting. (P111, 47 years)

Patients reported that the most common side effects of chemotherapy treatment included gastrointestinal issues that impacted their ability to consume food.

#### 3.3.2 Subtheme 1.2: Chronic Health Issues

Participants reported chronic symptoms including a low white blood cell count, severe bodily pain, fatigue, and hair loss. The loss of hair presented a significant challenge to the woman's self‐perception, as several participants felt grief over the hair loss caused by chemotherapy.

As soon as I had my fifth chemotherapy, I felt like I was slowly losing my life. All the hair on my head fell out. All my nails were black, but now they are much better. I had pain in my head, arms, legs, eyes, teeth, feet, nails, everywhere. There was no part of the body where pain was not present. (P134, 44 years)

When my four sessions of chemotherapy were completed, I became extremely ill. I was very fatigued. No medicine was giving me relief. Then, the doctors told me that my white blood cells were deficient. The doctor said that my survival was challenging, and if I had not been admitted to the hospital, my survival would have been tough. I was feeling severe pain in my body. (P140, 55 years)

Patients complained that chronic experiences, such as low immunity, pain, and fatigue, were uncomfortable and adversely impacted the individual's autonomy.

#### 3.3.3 Subtheme 1.3: Impact on Daily Life

Most patients (*n* = 34) stated they faced substantial limitations in their everyday tasks, and chemotherapy negatively affected their quality of life. However, one of the primary challenges they faced was the inability to manage their regular household duties and routine tasks, as well as the diminished ability to care for their children as they had previously done. Certain employees discontinued their jobs due to chemotherapy and expressed being unable to perform their office responsibilities, resulting in their decision to seek leave.

I find it difficult to do housework. I can't wash my clothes. I only do minor housework, like making curry. Ugh, I'm so tired. The most challenging task for me during chemotherapy was taking care of my children. (P103, 47 years)

Chemotherapy has changed my daily life. I had to take leave from the office. After 20 days, chemo starts again. Anyway, the ten days after chemo are very difficult. So I won't be able to go to the office for ten days. (P113, 36 years)

Participants reported that chemotherapy challenged them to manage routine duties and professional commitments, significantly disrupting their lives.

### 3.4 Theme 2: Psychological Impact

#### 3.4.1 Subtheme 2.1: Emotional Distress

The primary psychological consequence of chemotherapy was a higher incidence of negative emotions. More than half of breast cancer patients (*n* = 22) having chemotherapy experienced feelings of depression and a reduction in their ability to be individualized and autonomous.

I was worried at that time and was in depression. I had no hope that I would recover. (P110, 42 years)

When I found out that I had chemotherapy for cancer, I was afraid that I was going to die. I have had negative thoughts in my mind. And I'm still scared because cancer patients do not live long. I'm still afraid. (P118, 54 years)

Participants experienced psychological distress that was consequently linked to more intense levels of negative thought, depression, and anxiety. Patients thought that it would be challenging for them to live with illness.

#### 3.4.2 Subtheme 2.2: Refusal to Accept Illness

The confirmation of cancer significantly affects those with the illness. A minority of patients (*n* = 4) showed refusal of their cancer diagnosis. It requires some time to come to terms with the fact that they are patients.

When the doctor told me I had cancer, I had tears in my eyes. I said that I came from home only to take medicine. I did not get any more treatment. I can't have cancer. (P136, 50 years)

When the doctors told me that I had cancer, I couldn't believe it. I said I had nothing. How could I have cancer? I got my tests done again. I had two biopsies. A biopsy was done again at my request. Then, I was convinced that I had cancer. (P120, 40 years)

Individuals had heightened levels of fear and nervousness upon learning their diagnosis, and some of them requested verification of cancer through repeated tests.

#### 3.4.3 Subtheme 2.3: Fear of Recurrence

Despite improvements in healthcare, some individuals still have fears of recurrence. The participants (*n* = 17) expressed concerns regarding their future health; they believed that cancer recurrence was rather prevalent, even among individuals who had finished their medical treatment.

This disease is said to be silent and can recur. It can happen again in any part of the body, such as the kidney, bladder, etc. I take proper care of my health and should come to the hospital whenever the doctors call me for a checkup, because nothing is more important than health. (P125, 42 years)

Even after chemotherapy, you should continue to have regular checkups because most people who have cancer once have it recur. When I was doing my cancer tests, etc., I saw many patients who had cancer relapses. They had an operation and chemotherapy, but then they got cancer in the other breast. (P107, 49 years)

Some participants considered that cancer could recur after the chemotherapy, prompting them to perform regular checkups.

#### 3.4.4 Subtheme 2.4: Social Isolation

Many participants (*n* = 19) prevented gatherings such as birthdays and weddings to avoid questions about the changes occurring in their bodies. These changes in their physical appearance led them to confine themselves, seeking to avoid the distress caused by the negative reactions of others.

If I go somewhere, people repeatedly ask what happened. How much treatment is going on? People are constantly reminded of the disease, so patients remain in a state of tension. I did not meet people because of their questions. My health was not so good, so I avoided people. (P110, 42 years)

After the first session of chemotherapy, all my hair fell out. Physical change also affects you a lot. I started hiding from people because I didn't have hair. I had a lot of problems in the beginning. But gradually, I strengthened myself. (P140, 55 years)

Chemotherapy induces physiological alterations in the bodies of almost all patients. Participants reported isolating themselves from others to prevent their distressing responses and inquiries regarding their looks.

### 3.5 Theme 3: Economic Strain

#### 3.5.1 Subtheme 3.1: Treatment Cost

In this study, most participants (*n* = 33) reported economic difficulties as a highly significant barrier encountered throughout the treatment process. Patients struggled with limited resources, with some resorting to the sale of expensive assets to secure the money necessary for treatment.

I got scared because the treatment was expensive. The disease was not bothering me so much as it was difficult for me to afford it. (P120, 40 years)

Cancer treatment was beyond my reach. We used to spend a lot. We could not afford it, so we sold our house, land, and everything. And I got treated with this money. (P138, 45 years)

Most patients mentioned constrained financial resources, making it difficult to afford such costly treatment.

#### 3.5.2 Subtheme 3.2: Transportation Issues

Transportation is a significant cost. A certain number of patients (*n* = 17) from rural settings typically travel very long distances to healthcare facilities. Due to the high transportation costs, these individuals have difficulty attending appointments. Many individuals lack access to personal transportation, thus traveling to high‐priced cabs or public buses.

There should be concessions in the treatment of poor people. It's not just chemo expenses; there's much more to it. The cost of transportation to the hospital is also very high, and it was challenging for me to afford it all. (P111, 47 years)

It was complicated for me to afford the cost of transportation. You cannot travel on local transport when you are sick. You need a separate car to get to the hospital, which is very expensive. (P140, 55 years)

Patients experienced challenges in affording their transportation and logistics needs. They reported that while feeling ill, they need separate cars to reach the hospital, exacerbating their financial burden.

#### 3.5.3 Subtheme 3.3: Diet and Nutrition Expenses

The financial burden of cancer treatment and the necessity of a nutritious diet are significant. A balanced diet is crucial for good health, especially during cancer treatment. Women (*n* = 7) reported that they were unable to afford it; they revealed that chemotherapy, along with other expenses, made it challenging for them to maintain a healthy and balanced diet.

The patient's diet should also be well‐balanced with chemo, so the cost increases even more. No fruit is cheap. How expensive is a good diet? By combining all these things, the financial burden on the patient becomes substantial. (P120, 40 years)

Patients complained that higher medical expenses, when combined with the additional cost of a balanced diet, led to insignificant out‐of‐pocket costs, imposing a heavy burden on them.

### 3.6 Theme 4: Informational Challenges

#### 3.6.1 Subtheme 4.1: Lack of Prior Knowledge About Chemotherapy

A large number of patients (*n* = 25) had limited knowledge of chemotherapy. They were not aware of the administration of chemotherapy and its side effects. They only knew that the treatment would kill cancerous cells in their bodies.

At first, I didn't know about chemotherapy. I thought chemotherapy would be like kidney dialysis. I had that in mind. But when chemotherapy started, it seemed like a drip. (P130, 54 years)

I didn't know much about chemotherapy. However, I searched on the Internet about the possible side effects of chemotherapy. I had searched everything, and after chemotherapy, the same thing happened to me. (P140, 55 years)

Most participants who lacked information about treatment searched on the Internet and also obtained knowledge from relatives.

#### 3.6.2 Subtheme 4.2: Lack of Pretreatment Guidance

Medical practitioners provided patients with limited information in the initial planning phase. However, participants (*n* = 18) reported that before the start of chemotherapy, they were not informed about the potential side effects and their handling and care upon occurrence. Instructing patients on dietary decisions and daily routines during treatment is essential for effective outcomes. Participants mentioned a lack of education regarding dietary choices and restrictions.

Doctors did not tell me anything before starting chemo and did not guide me on what to eat or not. I heard from women that chemo causes hair loss. (P109, 40 years)

My chemotherapy was about to start. But I had no idea about the side effects of chemotherapy. The doctor guided me a little. But in routine, doctors have many patients. That is why they cannot guide completely. It is a common practice among doctors. But for the patient, it is all new. (P140, 55 years)

Most of the knowledge that physicians have provided was not often explained. Several participants complained that the information delivered during appointments did not fulfill their particular needs or demands due to insufficient detail.

#### 3.6.3 Subtheme 4.3: Insufficient Doctor‐Patient Communication

Proper interactions between patients and physicians throughout treatment are essential for early identification of issues, crisis avoidance, psychological health, and improved patient support. In the present study, a limited number of participants (*n* = 8) did not communicate with their healthcare professionals due to stress and nervousness.

I never discussed any side effects with the doctors because I was very nervous and tense when I came to the hospital. I didn't even want to come here, so I didn't have any discussion with a doctor. (P110, 42 years)

Patients reported that they did not even share their personal experiences of side effects with their doctor. They also avoided communicating with the doctor due to a fear of chemotherapy.

### 3.7 Theme 5: Coping and Support Systems

#### 3.7.1 Subtheme 5.1: Alternative Treatments

Some patients (*n* = 14) stated using alternative therapies suggested by others alongside their chemotherapy regimen. These included herbs, lemon water, turmeric, black cumin, olive oil, and so forth.

I used herbs such as dhamasa, turmeric, and lemon water. (P113, 36 years)

I used some of the tips people were suggesting to me. I used dhamasa herb for some time. I also used hemp and black cumin seeds and felt better. (P139, 60 years)

Patients indicated that they observed an improvement in their illness following the use of herbal remedies.

#### 3.7.2 Subtheme 5.2: Management of Side Effects

A few individuals (*n* = 15) employed strategies to minimize the physical impacts of chemotherapy, such as applying henna to hide discolored nails and using makeup to enhance their physical appearance. Only a few went for morning walks. Most of them used scarves and caps when their hair fell out.

When my hair fell out, I used to wear scarf and cap to cover my head. (P110, 42 years)

When my nails were black. I applied henna to hide my black nails. (P115, 35 years)

During chemotherapy, I used to maintain myself. I go for morning walks and exercise to feel well … I used to come to the hospital with makeup so no one would consider me a patient. (P140, 5 years)

Participants reported that coping strategies helped them manage the side effects of chemotherapy.

#### 3.7.3 Subtheme 5.3: Stay Motivated

The strength and courage of patients are essential for survival in the challenging and prolonged struggle of breast cancer. Many patients (*n* = 18) revealed that they remained motivated throughout their medical treatment and determined to deal with their problems rather than hide from them.

When I was diagnosed with cancer, I was not afraid. Everyone in my family was crying a lot. And I was comforting them all. I motivate myself and my family. My family used to get upset after seeing me, but believe me, I have never had a single tear in my eyes. (P107, 49 years)

I am brave. I fought this disease with courage. (P119, 55 years)

The participants highlighted the significance of courage to survive, with the majority supporting their loved ones despite the challenging conditions.

#### 3.7.4 Subtheme 5.4: Support From Friends and Family

Support from spouses and other family members was commonly observed among most participants (*n* = 32). Respondents were further assisted by their friends and extended relatives.

My family is my inspiration. All my family members are very supportive; they don't care about anything else; they want me to have a healthy life. (P107, 49 years)

I received a lot of support from my husband. He was with me, taking care of me day and night. I couldn't do housework at all. My husband had many responsibilities, including cooking, taking me to the hospital, and, most importantly, doing his job. (P125, 42 years)

Most patients received familial support, which strengthened their courage in overcoming the difficulties of breast cancer and combating the illness.

## 4 Discussion

Breast cancer significantly affects women worldwide, encompassing a range of challenges, feelings, and triumphs. Upon diagnosis, women embark on a challenging path that strains their mental and physical willpower, determination, and support networks. This study aimed to investigate the chemotherapy‐related challenges and experiences encountered by Pakistani women. In‐depth interviews revealed detailed views of breast cancer patients and their coping strategies in the critical phase of chemotherapy treatment. Chemotherapy has affected each patient differently and to varying extents. Some of the patients stated having slight side effects, while others experienced severe side effects following chemotherapy [40]. In our study, women with breast cancer experienced hair loss, darkened nails, mouth sores, weakness, and vomiting as the most frequent side effects. The literature reports similar experiences, indicating that more than 50% of patients experience hair loss due to chemotherapy [15, 41]. Miller, Gorcey, and McLellan [42] reported that chemotherapy induces alterations in nails and skin that may adversely impact women's self‐confidence. Additionally, vomiting has been observed as one of the common disturbing side effects, as shown by various studies [16, 43]. The resulting consequences disrupted their everyday routine and housework, affecting their jobs and duties as parents. In contrast, some women prefer to engage in activities that distract them and avoid thinking about the side effects [44]. Due to the high incidence of physical challenges, assistance from oncology pharmacists related to the side effects of chemotherapy is required to improve their overall well‐being and health.

The study's findings revealed that individuals experienced psychological effects that substantially affected their emotional health, leading to anxiety and depression. This agreed with the findings of other research, indicating that numerous psychological issues were linked to breast cancer [16]. Some women showed a fear of recurrence even after treatment was completed. Following our research's responses, Zhang et al. [45] indicated that patients who completed their treatment experienced a fear of recurrence linked to an increased risk of death, prompting them to undergo regular examinations and empower themselves to deal with their health problems. In our study, females diagnosed with breast cancer indicated experiences of social isolation. The respondents hesitated to visit to avoid questions about the changes affecting their physical appearance. This conclusion agreed with the literature indicating that body alteration limited their participation in social gatherings [11]. These results recommend that patients require continued medical monitoring by healthcare practitioners throughout and after treatment to control stress, depressive disorders, and social isolation of patients, which could enhance the level of their ease.

The present study addressed the significant financial burden of healthcare expenses on most participants. They especially highlighted the economic difficulties they faced in paying for medications, transportation for follow‐up visits, a proper diet, and related expenses. Additional studies have also revealed economic strain as a challenge to the early detection of breast cancer [46, 47]. The study participants reported difficulties in taking drastic measures, such as selling their house, to pay for therapy expenses. Related experiences were documented in another study, where individuals had to sell off their assets, such as jewelry and property, and incur loans to finance medical costs [48]. A systematic review by Pisu et al. [49] indicated that financial problems could impact the daily lives of most patients. Patients and their family members considered it challenging to manage the overall burden. They raised concerns about the expenses associated with cancer treatment. Both government and nongovernmental organizations should urgently establish strategies that offer financial services to support patients in managing the economic cost of their medical treatment. This need for financial support is not only crucial but also a shared responsibility of all those involved in healthcare.

Each participant had varying levels of information related to breast cancer chemotherapy. Unfortunately, the present study indicates that many participants had inadequate knowledge regarding chemotherapy and its related side effects. Patients were also provided with minimal chemotherapy‐related guidance from healthcare professionals because of limited time for consults and hectic schedules. This result correlates with a previously conducted study showing that respondents were not sufficiently educated and informed about chemotherapy‐related side effects prior to the initiation of the treatment, which negatively impacted their overall quality of life [11]. Effective communication between physicians and patients is essential for providing quality health treatment. However, women experience anxiety and hesitate to enquire further about their health condition and the course of treatment. Related research on communication between breast cancer patients and physicians revealed that communication issues were more prevalent among patients. The patient reported challenges with communication related to heightened fear, confusion, hopelessness, and anxiety in the follow‐up session [50]. These results suggest that healthcare providers should provide patients with relevant details of chemotherapy before it is started to promote knowledge. Facilitating a productive relationship between patients and doctors helps patients be well informed about any issues associated with chemotherapy.

Most participants lacked knowledge of managing the physical and psychological consequences. However, only a few patients could efficiently minimize the consequences of these side effects by using alternative therapies. The existing literature shows the prevailing trust among cancer patients in alternative treatments [51, 52]. The present study revealed that most participants suffered from hair loss, which led them to wear caps and scarves. They also improved their self‐care by engaging in physical exercise and applying cosmetics to look fresher. According to a relevant study, almost all females experience hair loss, a highly fearful consequence. Thus, they decided to use scarves and wigs to conceal their bald heads, and a few have also acquired expertise in applying makeup [53]. Further, adopting self‐care practices, continuing determination, and receiving support from friends and family can significantly contribute to managing and coping with side effects. Such coping methods are consistent with Lazarus and Folkman's coping theory, which clarifies both problem‐focused strategies (e.g., alternative treatments, self‐care procedures) and emotion‐focused strategies (e.g., emotional support from friends and family, sustaining motivation) [54].

The study also highlights the crucial role of friends and family as the primary patient support providers. Their support provided them with strength and determination, which complies with social support theory. It indicates that emotional, informational, and practical support from friends and family assists individuals in coping with stress and positively impacts their overall health [55, 56]. Prior research also showed friends and family support for cancer patients, which strengthened their courage in dealing with the difficulties of breast cancer, achieving favorable treatment results, and combating cancer [57]. Oncology pharmacists provide patients with appropriate assistance and encouragement to support them as they face the challenges of chemotherapy. Their role can be institutionalized by incorporating oncology pharmacists into multidisciplinary collaborations, facilitating their contributions to side‐effect management, patient care, and overall treatment coordination. Implementing specialized training programs with continuous reinforcement approaches can ultimately improve outcomes for patients in oncology contexts across Pakistan [58, 59].

## 5 Implications and Future Research

The outcomes of our study emphasize the critical need to ensure that health systems are ready to support the treatment of breast cancer as well as various long‐term illnesses. We expect this exploratory qualitative research to offer novel perspectives, a favorable context, and significant research on breast cancer chemotherapy in females. A vital advantage of this research is that it contributes to a limited but probably expanding library of literature aimed at providing high‐quality medical care for breast cancer patients. This study may serve as an informative tool for health care providers to address the challenges of breast cancer chemotherapy in women. The outcomes of this research may inform improvements in chemotherapy‐related health services by highlighting the key considerations that result from patients' experiences, as summarized in Table 4. By enhancing knowledge about cancer and its management, this research enables patients to identify and better cope with chemotherapy‐related complications. It also helps caregivers implement patient‐centered strategies, provides psychological counseling, and offers communication techniques that can enhance treatment outcomes and strengthen patient confidence. This study identifies some aspects that offer significant potential for future research throughout many settings, exploring these themes in broader populations and how women from various backgrounds cope with the financial strain and long‐term consequences of chemotherapy treatment.

**TABLE 4:** Summary of consideration for conducting breast cancer chemotherapy‐related research among females.

Topics	Considerations
Physical side effects of chemotherapy	To reduce side effects and improve patients' quality of life, guidance from medical professionals and proactive efforts, including adequate self‐care, dietary habits, and encouragement, should be integrated
Psychological impact	Patients require emotional support and regular medical supervision from healthcare professionals during treatment to manage stress, depressive conditions, and anxiety, which could control their psychological issues
Support systems	The support of friends and family strengthened cancer patients' motivation to deal with the mental and physical difficulties of breast cancer treatment. Healthcare practitioners' support and encouragement also assist patients in fighting the struggles of chemotherapy
Informational gaps	Maintaining positive interactions between patients and physicians minimizes communication gaps, which is crucial for delivering quality healthcare
Economic strain	Financial support services and government interventions are required to improve treatment affordability and reduce patients' economic burdens
Post‐chemotherapy care	Follow‐up assessments are crucial for patients to deal with post‐chemotherapy side effects and facilitate their return to daily life routines

## 6 Limitations

The current study has some limitations. First, this study was carried out on Pakistani individuals. Hence, certain features may not accurately represent the personal experiences of breast cancer individuals from countries other than Pakistan. Second, the study's findings are not extended, as it was performed exclusively at PIMS. The outcomes can be restricted to this particular facility. Third, data findings do not include the experiences of males with breast cancer, while a study involving this demographic may significantly improve the area of research. Fourth, we have never gathered data from hospitalized patients or those suffering physical distress. Fifth, the age range of the respondents was between 30 and 70 years, which does not represent the experiences of women affected with cancer at much younger ages. Sixth, a greater percentage of the subjects were married, had low academic backgrounds, were unemployed, and had low family incomes. These findings may not apply to all individuals. Lastly, as all participants were interviewed after completing chemotherapy sessions, their responses may have been affected by recall bias. Irrespective of all these limitations, we desire that our research findings contribute to the comprehension of women's experiences with breast cancer.

## 7 Conclusion

This study highlights that women having chemotherapy for breast cancer experience significant physical side effects, psychological impacts, financial strain, and insufficient information related to chemotherapy before their treatment begins, which are serious challenges for many females. This study implies that healthcare providers promote patient outcomes by addressing physical and psychological consequences and providing comprehensive information to patients regarding chemotherapy and the possible side effects before chemotherapy begins. Oncology pharmacists have the expertise to guide patients on suspected side effects, their management, and chemotherapy administration. Moreover, both government and nongovernment organizations should provide financial aid for costly treatments, and an economic assistance program for patients with limited income is essential to lighten the financial burden on struggling people and their families.

## Author Contributions

Conceptualization: Rehana Sarwat, Ali Ahmed, and Maryam Mahmood. Data curation: Rehana Sarwat, Ali Ahmed, and Ayesha Azam. Formal analysis: Rehana Sarwat, Ali Ahmed, Zakir Khan, Muhammad Amir Hamza, Bushra Batool Zahra, and Ayesha Azam. Investigation: Rehana Sarwat and Bushra Batool Zahra. Methodology: Rehana Sarwat, Ali Ahmed, Maryam Mahmood, Muhammad Amer, and Bushra Batool Zahra. Project administration: Rehana Sarwat, Ali Ahmed, Maryam Mahmood, and Muhammad Amir Hamza. Resources: Zakir Khan, Muhammad Amir Hamza, Bushra Batool Zahra, and Ayesha Azam. Supervision: Ali Ahmed. Validation: Ali Ahmed and Muhammad Amer. Writing – original draft: Rehana Sarwat. Writing – review and editing: Rehana Sarwat, Ali Ahmed, Zakir Khan, Maryam Mahmood, Muhammad Amir Hamza, Muhammad Amer, Bushra Batool Zahra, and Ayesha Azam.

## Ethics Statement

This study has obtained ethical approval from the Research Ethics Committee of Riphah Institute of Pharmaceutical Sciences, Riphah International University (Ref. No. REC/RIPS/2023/26) and PIMS Hospital Islamabad, and from its associated educational facility, Shaheed Zulfiqar Ali Bhutto Medical University (SZABMU) (No. F.1‐1/2015/ERB/SZABMU/1234).

## Consent

The participants gave written informed consent to participate in this research study and signed the consent agreement.

## Conflicts of Interest

Zakir Khan is supported by the European Union through the Marie Sklodowska‐Curie Actions (MSCA) programme (Project acronym: HEAD‐P; Project number: 101149577). Ali Ahmed received support from the U.S. National Institute of Mental Health (NIMH) RO1MH126768 and R21MH132406. The other authors declare no conflicts of interest.

## Supporting information


**Data S1:** cnr270381‐sup‐0001‐supinfo.docx.

## Data Availability

The data that support the findings of this study are available in the Supporting Information of this article.
