# Supplementary material for: Exploring Challenges Related to Breast Cancer Chemotherapy Among Females in Pakistan: A Qualitative Study
Source: Cancer Rep (Hoboken). 2025 Nov 13;8(11):e70381. doi: 10.1002/cnr2.70381 (PMC12614083; doi:10.1002/cnr2.70381)
Supplement: Supplementary file 1 — Data S1: cnr270381‐sup‐0001‐supinfo.docx. [file CNR2-8-e70381-s001.docx]

**Supplementary Table 1: COREQ (COnsolidated criteria for REporting Qualitative research) checklist**

| **No. Item** | **Guide questions/description** |  | **Location in**  **Manuscript (Section)** |
| --- | --- | --- | --- |
| **Domain 1: Research**  **team and reflexivity** |  |  |  |
| *Personal Characteristics* |  |  |  |
| 1. Inter viewer/facilitator | Which author/s conducted the interview or focus group? | Rehana Sarwat and assist by Ali Ahmed | Author Contributions |
| 2. Credentials | What were the researcher’s credentials? E.g. B.A, MPhil,  PhD | M.Phil. PhD | Title page |
| 3. Occupation | What was their occupation at the time of the study? | Researcher and MPhil student in Pharmacy Practice | - |
| 4. Gender | Was the researcher male or  female? | Interviewer was female | - |
| 5. Experience and training | What experience or training did the researcher have? | Training in qualitative  research methodologies | - |
| *Relationship with*  *participants* |  |  |  |
| 6. Relationship established | Was a relationship  established prior to study commencement? | Yes | - |
| 7. Participant knowledge of the interviewer | What did the participants know about the researcher?  e.g. personal goals, reasons for doing the research | Before starting the interview, participants were informed about the study's purpose and confidentiality | Methodology |
| 8. Interviewer characteristics | What characteristics were reported about the inter viewer/facilitator? e.g. Bias, assumptions, reasons and interests in the research topic | Research conducted for academic purposes; no bias identified | - |

| **Domain 2: study design** |  |  |  |
| --- | --- | --- | --- |
| *Theoretical framework* |  |  |  |
| 9. Methodological orientation and Theory | What methodological orientation was stated to underpin the study? e.g. grounded theory, discourse analysis, ethnography, phenomenology, content  analysis | Thematic analysis | Methodology |
| *Participant selection* |  |  |  |
| 10. Sampling | How were participants selected? e.g. purposive, convenience, consecutive, snowball | Purposive sampling | Methodology |
| 11. Method of approach | How were participants approached? e.g. face-to-face,  telephone, mail, email | All interviews were conducted face-to-face | Methodology |
| 12. Sample size | How many participants were  in the study? | 40 participants | Methodology and  Results |
| 13. Non-participation | How many people refused to participate or dropped out?  Reasons? | None recorded | - |
| *Setting* |  |  |  |
| 14. Setting of data collection | Where was the data collected? e.g. home, clinic, workplace | Participants were interviewed at PIMS | Methodology |
| 15. Presence of non- participants | Was anyone else present besides the participants and  researchers? | No | - |
| 16. Description of sample | What are the important  characteristics of the sample?  e.g. demographic data, date | Demographics | Results |
| *Data collection* |  |  |  |
| 17. Interview guide | Were questions, prompts, guides provided by the authors? Was it pilot tested? | An interview guide was based on a literature review. It was pilot-tested on four women | Methodology |
| 18. Repeat interviews | Were repeat interviews carried out? If yes, how many? | No | - |
| 19. Audio/visual recording | Did the research use audio or visual recording to collect the  data? | Interviews were audio recorded | Methodology |

| 20. Field notes | Were field notes made during  and/or after the interview or focus group? | During the interviews, relevant notes were taken | Methodology |
| --- | --- | --- | --- |
| 21. Duration | What was the duration of the inter views or focus group? | Each interview was 15 to 20 minutes long | Methodology |
| 22. Data saturation | Was data saturation  discussed? | Yes | Methodology |
| 23. Transcripts returned | Were transcripts returned to participants for comment and/or correction? | No | - |
| **Domain 3: analysis and**  **findings** |  |  |  |
| *Data analysis* |  |  |  |
| 24. Number of data coders | How many data coders coded the data? | All the authors except Muhammad Amer and Maryam Mahmood  participated in data coding | Authors’ contributions and Methodology |
| 25. Description of the coding tree | Did authors provide a  description of the coding tree? | Yes | Methodology |
| 26. Derivation of themes | Were themes identified in advance or derived from the data? | Themes were derived from the data | Methodology |
| 27. Software | What software, if applicable,  was used to manage the data? | None | - |
| 28. Participant checking | Did participants provide  feedback on the findings? | No | - |
| *Reporting* |  |  |  |
| 29. Quotations presented | Were participant quotations presented to illustrate the themes/findings? Was each quotation identified? e.g. participant number | Yes | Results |
| 30. Data and findings consistent | Was there consistency  between the data presented and the findings? | Yes | Results and Discussion |
| 31. Clarity of major  themes | Were major themes clearly  presented in the findings? | Yes | Discussion |
| 32. Clarity of minor themes | Is there a description of diverse cases or discussion of  minor themes? | Yes | Discussion |

**Supplementary Table 2. Patient Additional Quotes Highlighting Challenges Related to Breast Cancer Chemotherapy**

| **Themes** | **Sub Themes** | **Patient Additional Quotes** |
| --- | --- | --- |
| **Physical challenges** | Acute physical effects | One of the most common side effects of chemotherapy that I experienced was vomiting. Even drinking water became very difficult for me. I also had stomach problems, which is why I could not eat anything. **(P103, 47 years)**  I vomit a lot. Whenever I saw the food, I immediately vomited. Everything smells bad. **(P115, 35 years)** |
|  | Chronic health issues | All my hair fell out because of the chemo. I was very depressed because hair is a part of your body. The biggest challenge that I faced was hair loss. **(P115, 35 years)**  All my hair fell out. I'm only worried about my hair, nothing else. When my hair fell, I cried when I saw it (laughs). It was painful for me. My kids said not to worry about it and suggested to shave my head. Because when a woman sees her hair falling, it is very unbearable for her. My hair was very healthy and beautiful. That time was painful for me. **(P119, 55 years)** |
|  | Impact on daily life | Chemotherapy has many side effects. Chemotherapy made my life very difficult. In illness, it becomes difficult to do housework and manage everything. **(P127, 38 years)**  Because of the chemotherapy, I suffered a lot; my health was not good. There was repeated vomiting. I could not do any housework. **(P134, 44 years)**  I had a hard time during the chemotherapy. I could not manage the housework. I was encouraged to see other people, and if other people can be cured, why not me? My husband supported me a lot. He took great care of everything. **(P137, 45 years)** |
| **Psychological impact** | Emotional distress | When I came for chemo, every time, I had mental pressure that I had to undergo chemo. I get tensed immediately. There was a lot of fear and dread. **(P108, 49 years)**  I was better before. But now I am afraid that if something happens to me, what will happen to my children? Now, I have negative thoughts.  I feel sad, and often, I start crying when I have depressive thoughts. Because of this, my children get upset. My house gets disturbed. **(P139, 60 years)** |
|  | Refusal to accept illness | The doctor did my biopsy. After the biopsy, the doctor said that first, we would give you chemotherapy. But I was not accepting that I had cancer**. (P113, 36 years)** |
|  | Fear of recurrence | When I come to the hospital, I meet people. So people say someone gets back cancer after five years, someone after six years. Therefore, it is important to perform regular checkups. I was afraid of recurrence. **(P114, 62 years)** |
|  | Social isolation | I did not attend weddings. It's not that I didn't have the courage. If I wanted to go, I could. But I used to think that now I don't have hair, eyebrows, or eyelashes. I feared that people would see me and say I was a patient. So, to avoid these sentences, I did not go to gatherings. People sympathize with me, but I don't feel good. **(P120, 40 years)**  When I had chemotherapy, I went to my room. I didn't like to meet people. I didn't even have the strength to face them. **(P121, 42 years)** |
| **Economic strain** | Treatment cost | Money was a significant issue in my treatment. It wasn't easy to afford such an expensive treatment. We are middle-class people. Our finances are very weak. **(P103, 47 years)**  I was worried about how I would be able to afford such an expensive treatment. And if not treated, I will die. **(P106, 40 years)**  Hospital visits, operations, tests, chemotherapy, and all these things cost a lot. Chemotherapy alone is not enough. There are many other expenses. It is complicated for a poor man to afford**. (P132, 63 years)** |
|  | Transportation issues | Transportation was also a problem during treatment. I couldn't afford to visit the hospital by taxi because taxi drivers charge a lot of money. **(P103, 47 years)**  My treatment has cost a lot of money. And it was difficult to pay the car rent again and again. The car rental was also costly. **(P115, 35 years)** |
|  | Diet and nutrition expenses | The monthly expenses become very high. You must also maintain your diet and take all your medicines. If your diet is not good, you will become weaker. If you manage your health, it becomes costly**. (P113, 36 years)** |
| **Informational challenges** | Lack of prior knowledge about chemotherapy | Before the chemotherapy started, I only had the idea that the hair would fall out. I didn't know the actual pain and side effects. **(P110, 42 years)**  I didn't know much about cancer treatment. No one in our family had cancer. **(P121, 42 years)** |
|  | Lack of pre-treatment guidance | Doctors did not inform me about side effects or anything else.….. It is very important to guide patients about disease. Even the few side effects I experienced would not have occurred if doctors had adequately guided me. **(P107, 49 years)** |
|  | Insufficient doctor-patient communication | When I had side effects of chemotherapy, I did not discuss them with the doctor. I was very afraid at that time. **(P103, 47 years)** |
| **Coping and Support Systems** | Alternative treatments | I followed all the remedies that people suggested, like dhamasa herb (fagonia arabica) and sumbul (musk root). I also used turmeric in olive oil. And still, I use a lot of turmeric (laughs). **(P108, 49 years)**  I took dhamasa herb for some time, then stopped. Now I eat black cumin, because black cumin cures every disease except death. **(P132, 63 years)** |
|  | Management of side effects | I had no hair, so I wore a scarf. I didn't use any artificial things or wigs. **(P108, 49 years)** |
|  | Stay motivated | I don't need anyone. I do all my work and household work myself. Even doctors are amazed at my courage. I am very brave. I used to come alone for chemo. When I was undergoing chemo, there were thousands of patients in the hospital. And everyone had 3 or 4 people with them. But I am courageous. I used to go for chemo alone. **(P104, 55 years)**  I used to signal to my body that I would be fine. This helped me a lot. We all know that we will die one day, but it is very important to motivate yourself. I now guide the cancer patients around me. I like to help others, and I share my experience with others to help others. **(P140, 55 years)** |
|  | Support from friends and family | During this time, my parents were my biggest support. They looked after me 24 hours a day and encouraged me a lot. My brothers also supported me. My in-laws and my husband also cooperated a lot. My entire family was very supportive**. (P103, 47 years)**  My children supported me. They cared for me a lot. Half of the disease resolves if the patient is supported at home. But having no one to care for at home is very difficult. **(P137, 45 years)** |
